# The Counteracting Effects of Exercise on High-Fat Diet-Induced Memory Impairment: A Systematic Review

**DOI:** 10.3390/brainsci9060145

**Published:** 2019-06-20

**Authors:** Paul D. Loprinzi, Pamela Ponce, Liye Zou, Hong Li

**Affiliations:** 1 Exercise & Memory Laboratory, Department of Health, Exercise Science and Recreation Management, The University of Mississippi, Oxford, MS 38677, USA; pdloprin@olemiss.edu (P.D.L.); pponce@uthsc.edu (P.P.); 2 Shenzhen Key Laboratory of Affective and Social Cognitive Science, College of Psychology and Sociology, Shenzhen University, Shenzhen 518060, China; liyezou123@gmail.com; 3 Research Centre of Brain Function and Psychological Science, Shenzhen University, Shenzhen 518060, China; 4 Shenzhen Institute of Neuroscience, Shenzhen University, Shenzhen 518060, China

**Keywords:** cytokines, hippocampal neurogenesis, inflammation, insulin resistance, obesity

## Abstract

The objective of the present review was to evaluate whether exercise can counteract a potential high-fat diet-induced memory impairment effect. The evaluated databases included: Google Scholar, Sports Discus, Embase/PubMed, Web of Science, and PsychInfo. Studies were included if: (1) an experimental/intervention study was conducted, (2) the experiment/intervention included both a high-fat diet and exercise group, and evaluated whether exercise could counteract the negative effects of a high-fat diet on memory, and (3) evaluated memory function (any type) as the outcome measure. In total, 17 articles met the inclusionary criteria. All 17 studies (conducted in rodents) demonstrated that the high-fat diet protocol impaired memory function and all 17 studies demonstrated a counteracting effect with chronic exercise engagement. Mechanisms of these robust effects are discussed herein.

## 1. Introduction

Unlike traditional advice that promotes a low-fat diet [1], recently, high-fat diets (HFDs) are gaining popularity among athletes [2] and the general population [3]. However, HFDs have been shown to impair episodic memory function [4,5]. In humans, episodic memory function refers to the retrospective recall of information from a spatial-temporal context [6]. That is, episodic memory, a contextual-based memory, involves what, where, and when aspects of a memory [7]. In rodents, however, episodic memory is primarily evaluated from a spatial memory task, such as the Morris water maze task or a T-maze task.

As discussed elsewhere [8], a cellular correlate of episodic memory is long-term potentiation (LTP), a form of activity-dependent plasticity that results in enhancement of synaptic transmission [9]. The complementary process of LTP is long-term depression (LTD), in which the efficacy of synaptic transmission is reduced [10]. It is thought that LTP and LTD play an important role in memory as LTP- and LTD-like changes in synaptic strength occur as memories are formed at various sets of brain synapses [11,12,13]. The adverse episodic memory effects from an HFD may, in part, occur through alterations in processes that influence synaptic transmission and production of plasticity-related proteins [14,15,16]. For example, research demonstrates that a chronic HFD impairs hippocampal dendritic spine density [17], induces astrocyte alterations [18], reduces expression of the NR2B subunit of NMDA receptors [19], decreases CREB expression [20], and reduces hippocampal BDNF production [21].

Of central interest to this review is whether exercise can counteract HFD-induced memory impairment. Such an effect is plausible for several reasons. We speculate that this counteracting effect may occur from exercise activating some of the neurophysiological pathways that are involved in episodic memory function (e.g., BDNF). Further, we speculate that exercise may counteract HFD-induced memory impairment by, not only activating these pathways, but by also inhibiting the downregulation of these pathways induced by HFD. First, chronic exercise has been shown to enhance episodic memory function [22] and LTP [23]. Chronic exercise may subserve episodic memory function via inducing neurogenesis [23,24] and altering LTP-related receptor (e.g., NMDA) structure and function [25,26].

Couched within the above, HFD may impair episodic memory and exercise has been shown to enhance episodic memory function. Further, exercise has been shown to regulate processes (e.g., LTP) that are impaired with HFD. Thus, the specific research question of this systematic review was to evaluate the extant literature to determine whether exercise can counteract a potential HFD-induced memory impairment effect. 

## 2. Methods

### 2.1. Computerized Searches

The evaluated databases included: Google Scholar, Sports Discus, Embase/PubMed, Web of Science, and PsychInfo [27]. Articles were retrieved from inception to 22 April 2019. The search terms, including their combinations, were: exercise, physical activity, diet, high-fat, memory, cognition, and cognitive function.

### 2.2. Study Selection

The computerized searches were performed separately by two authors and comparisons were made to render the number of eligible studies. Consensus was reached from these separate reviews. After conducting the searches, the article titles and abstracts were evaluated to identify applicable articles. Articles meeting the inclusionary criteria were retrieved and evaluated at the full text level.

### 2.3. Inclusionary Criteria

Studies were included if: (1) an experimental/intervention study was conducted, (2) the experiment/intervention included both an HFD and exercise group, and evaluated whether exercise could counteract the negative effects of an HFD on memory, and (3) evaluated memory function (any type) as the outcome measure. 

### 2.4. Data Extraction of Included Studies

Detailed information from each of the included studies were extracted, including the following information: author, subject characteristics, exercise protocol, diet protocol, temporal assessment of the exercise and diet protocols, memory assessment, whether the diet protocol impaired memory, whether exercise counteracted the diet-induced memory impairment, and evaluated mechanisms of this attenuation effect. 

## 3. Results

### 3.1. Retrieved Articles

The computerized searches identified 448 articles. Among the 448 articles, 430 were excluded and 18 full text articles were reviewed. Among these 18 articles, 1 was ineligible as it did not meet our study criteria. Thus, in total, 17 articles met the inclusionary criteria and were evaluated herein. 

### 3.2. Article Synthesis

Details on the study characteristics are displayed in Table 1 (extraction table). As shown in Table 1, all studies employed an exercise and diet paradigm in an animal model. The daily exercise protocol ranged from 6 weeks to 23 weeks. Similarly, the diet protocol ranged from 6 weeks to 23 weeks, with the majority of studies implementing an ad libitum diet consisting of 60% fat, 20% carbohydrate, and 20% protein. Across the 17 studies, there was variability on the temporal assessment of the exercise and diet protocols, consisting of either having both protocols occur concurrently or exercise occurring after the high-fat diet (treatment paradigm). Among the 17 studies, 10 implemented a concurrent model, whereas 7 implemented a treatment paradigm. Regarding the memory outcome, the majority of studies utilized a Morris water maze task, with others employing an avoidance task (e.g., passive or step-down) or a maze task (e.g., y-maze task, radial maze task, evaluated plus maze task). 

Regarding the effects of HFD on memory, all 17 studies demonstrated that the HFD protocol impaired memory function. Notably, in one study, this impairment effect only occurred among a subgroup of animals (adolescent mice) [28]. Similarly, all 17 studies demonstrated that chronic exercise engagement counteracted HFD-induced memory impairment. Notably, however, one study showed that this attenuation effect only occurred if the chronic exercise protocol occurred during the majority of the HFD period [29].

## 4. Discussion

The present review examines whether exercise can counteract HFD-induced memory impairment. Main findings from the present review are twofold: (1) chronic HFD robustly impairs memory function, and (2) chronic exercise engagement, occurring either concurrently or after the diet protocol, robustly counteracted HFD-induced memory impairment. This latter finding occurred among studies that employed various exercise protocols, such as voluntary access to a running wheel or forced exercise on a treadmill. Similarly, across these studies, the exercise protocol varied from 6 to 23 weeks. Further, various spatial-related memory tasks were employed across the evaluated studies. Despite these variations in the exercise protocols and memory tasks, exercise robustly counteracted HFD-induced memory impairment. 

A mechanism through which exercise may counteract HFD-induce memory impairment is likely through alterations in processes related to synaptic transmission and production of plasticity-related proteins. As thoroughly addressed elsewhere [43,44,45,46,47,48], LTP involves several phases, including early-LTP (E-LTP) and late-LTP (L-LTP) [47]. In brief, E-LTP, a protein synthesis-independent process, involves the activation of several kinases (e.g., PKA, CaMKII), which play a critical role in phosphorylating proteins and receptors (e.g., AMPA, NDMA), eventually potentiating synaptic transmission [47]. Endocytosis of such receptors, via, for example, phosphatase activity, may induce LTD [10]. In contrast to E-LTP, L-LTP, a protein synthesis-dependent process, involves gene expression and local protein synthesis via, for example, the TrkB receptor [47]. The following paragraphs link some of these processes to episodic memory function, how HFD impairs these processes, and how exercise influences these processes.

**Figure 1:**
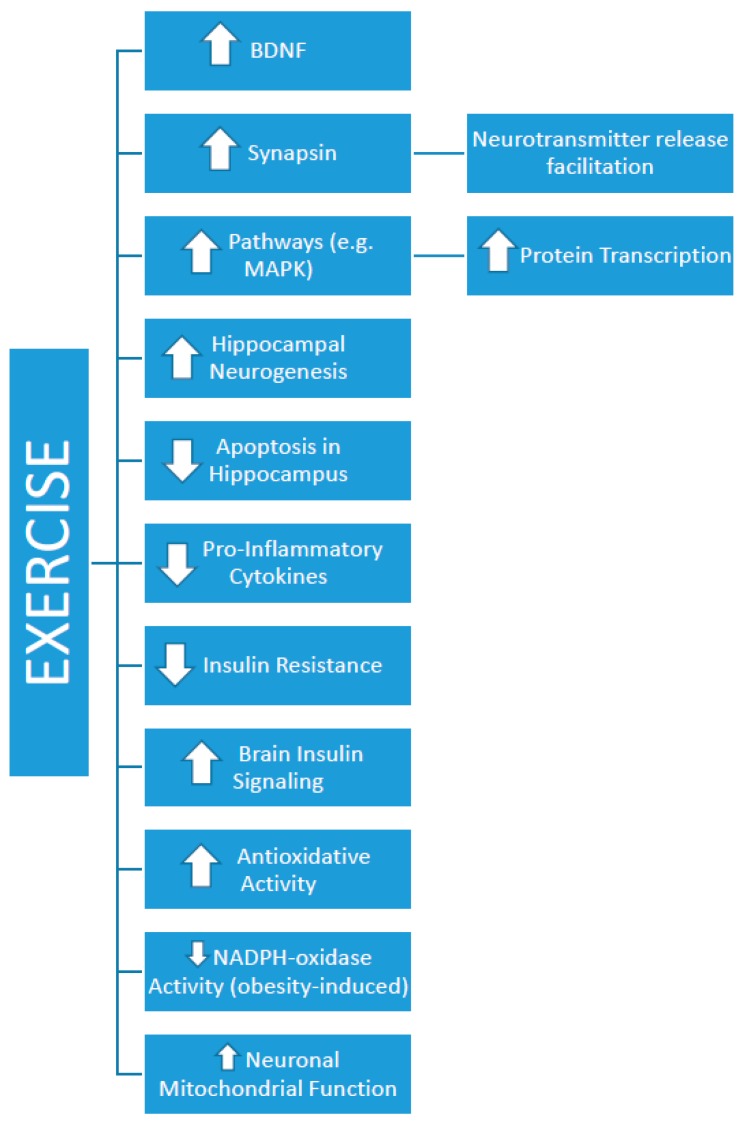
Schematic illustrating the mechanistic role through which exercise may counteract a high-fat diet-induced impairment of memory function.

As noted in Table 1 and as shown in Figure 1, potential mechanisms of this exercise-related counteraction effect of HFD-induced memory impairment are multifold. Such effects may include exercise-induced alterations in some of the above-mentioned pathways. For example, activation of the BDNF receptor, TrkB, plays an important role in spatial memory [49]. Specifically, BDNF appears to play a critical role in the consolidation of memories, as previous work demonstrates that continuous intracerebroventricular infusion of antisense BDNF oligonucleotide causes spatial memory deficit [50]. An HFD has been shown to reduce hippocampal BDNF levels and downstream effectors [20], which may lower the neurochemical substrate of the hippocampus that is needed for optimal neuronal function. Exercise may counteract this HFD-induced BDNF reduction and memory impairment via its role in augmenting BDNF levels, via β-hydroxybutyrate alteration [51]. Exercise-induced increases in β-hydroxybutyrate are thought to inhibit histone deacetylases, ultimately facilitating hippocampal BDNF expression [51].

In addition to BDNF, synapsin 1, a neuronal phosphoprotein, plays an important role in regulating neurotransmitter release. A chronic HFD has been shown to lower synapsin 1 levels [20] and reduction of synapsin 1 leads to spatial memory deficit [52,53]. Exercise has been shown to increase synapsin 1 levels [54], which is likely occurring from exercise-induced increases in BDNF (i.e., BDNF may promote the phosphorylation of synapsin 1) [55]. BDNF also plays an important role in hippocampal neurogenesis [56], which may play a causal role in spatial memory. Ablation of adult hippocampal neurogenesis results in impairment of acquiring spatial reference memory [57]. Neurogenesis plays an important role in spatial memory and may, for example, occur via pattern separation mechanisms [58]. A chronic HFD may impair neurogenesis through increases in corticosterone [59], with exercise potentially counteracting this effect via BDNF-mediated hippocampal neurogenesis [60].

In conclusion, this review demonstrated that episodic memory may be impaired with a chronic HFD, yet this effect may be counteracted by chronic engagement in exercise. Future work should consider this model in the context of a preventive paradigm. All of the evaluated studies in this review employed a concurrent or treatment-based model and, thus, it would be worthwhile to evaluate if a period of exercise prior to an HFD protocol can counteract the detrimental effects of an HFD on memory function. Furthermore, future work should also consider evaluating other memory systems (e.g., working memory, episodic memory, procedural memory, prospective memory) to determine whether the observed associations hold true across different memory systems. 

## Figures and Tables

**Table 1:** Extraction table of the evaluated studies.

Study	Subjects	Exercise Protocol	Diet Protocol	Temporal Assessment of Exercise and Diet	Memory Assessment	Did High-Fat Diet Impair Memory?	Did Exercise Counteract Diet-Induced Memory Impairment?	Mechanisms
Molteni et al. (2004) [4]	Fisher 344 rats, 2 months old	Free access to running wheel for 2 months.	2 months on high in saturated and monounsaturated fat (primarily from lard plus a small amount of corn oil, approx. 39% energy)	Concurrent	Morris water maze	Yes	Yes	Exercise reversed the decrease in BDNF and its downstream effector, synapsin I (involved in BDNF release). Exercise also increase CREB transcription.
Maesako et al. (2012) [5]	APP transgenic mice overexpressing the familial AD-linked mutation	Enriched environment with access to running wheel; this occurred from weeks 10–20 (i.e., 10 weeks into the high-fat diet).	20 weeks of high-fat diet, involving 60% fat, 20% CHO, and 20% protein	Concurrent	Morris water maze	Yes	Yes	Enriched environment attenuated diet-induced Aβ deposition.
Maesako et al. (2012) [30]	APP transgenic mice overexpressing the familial AD-linked mutation	Voluntary access to running wheel.	20 weeks of high-fat diet, involving 60% fat, 20% CHO, and 20% protein	Concurrent	Morris water maze	Yes	Yes	Exercise attenuated diet-induced Aβ deposition and strengthened the activity of neprilysin, the Aβ-degrading enzyme.
Maesako et al. (2013) [29]	APP transgenic mice overexpressing the familial AD-linked mutation	Voluntary access to running wheel.	20 weeks of high-fat diet, involving 60% fat, 20% CHO, and 20% protein	Concurrent	Morris water maze	Yes	Yes, but only if the exercise occurred throughout the majority of the diet protocol	
Woo et al. (2013) [31]	4-week-old Sprague–Dawley rats	Treadmill exercise for the first 8 weeks, involving a progressive exercise program, ranging from 40 to 60 min/day of exercise.	13-weeks of high-fat diet, involving 45% fat	Concurrent	Morris water maze	Yes	Yes	Upregulation of BDNF and MAPK.
Noble et al. (2014) [32]	7-month-old Naïve rats	Forced treadmill or voluntary wheel access for 7 weeks	16 weeks of high-fat diet	Exercise occurring after high-fat diet (treatment)	Two-way active avoidance test	Yes	Yes	Increased BDNF in CA3.
Cheng et al. (2016) [33]	Twelve-week-old C57BL/6J mice	Treadmill running, 60 min/day, 5 times/week, 15 m/min, for 16 weeks.	16 weeks of high-fat diet ad libitum, involving 60% fat, 20% CHO, and 20% protein	Concurrent	Morris water maze	Yes	Yes	p-CREB, BACE1, IDE, and PSD95 were likely mediators of this effect.
Kang et al. (2016) [34]	Sprague–Dawley rats aged 8 weeks	Treadmill running, 30 min/day, 5 days/week, for 8 weeks.	High fat diet (60% fat) for 20 weeks	Exercise occurring after high-fat diet (treatment)	Passive avoidance task	Yes	Yes	Reduction in pro-inflammatory cytokines (TNF-α, interleukin-1β).
Kim et al. (2016) [35]	Male C57BL/6 mice, 4-weeks old	Treadmill exercise, ranging from 30 to 50 min/day; progressive over a 20-week period.	High-fat diet (60% fat) for 20 weeks ad libitum	Exercise occurring after high-fat diet (treatment)	Y-maze test and radial-8-arm maze test	Yes	Yes	Increased expression of BDNF and TrkB and enhanced cell proliferation.
Klein et al. (2016) [28]	Six-week-old female C57Bl/6N mice	Free access to running wheel.	12 weeks of high-fat diet, involving 60% fat, 20% CHO, and 20% protein	Concurrent	Morris water maze	Yes, but only in adolescent	Yes	Modulation of hippocampal neurogenesis.
Park et al. (2017) [36]	Male 4-week-old C57BL/6 mice	Treadmill exercise, 6 days/week, approx. 40 min/day, for 12 weeks.	20 weeks of high-fat diet, involving 60% fat ad libitum	Exercise occurring after high-fat diet (treatment)	Step-down avoidance task	Yes	Yes	Reduced insulin resistance, improved mitochondrial function, and reduced apoptosis in the hippocampus.
Cheng et al. (2018) [37]	Male 3-week-old SHR and normotensive Wistar–Kyoto rats	Swimming exercise for 6 weeks.	6 weeks of low-soybean oil diet	Concurrently	Morris water maze	Yes	Yes	Up-regulation of BDNF and NMDA-r expression.
Jeong et al. (2018) [38]	Sprague–Dawley rats aged 20 weeks	Treadmill exercise for 8 weeks, 30 min/day, 8 m/min, 5 days/week.	High-fat diet for 20 weeks	Exercise occurring after high-fat diet (treatment)	Water maze and passive avoidance tasks	Yes	Yes	Improved brain insulin signaling, inhibition of obesity-induced NADPH-oxidase activity.
Jeong et al. (2018) [39]	Sprague–Dawley rats aged 8 weeks	Treadmill exercise for 8 weeks, 30 min/day, 5 days/week, progressive intensity.	High fat diet for 20 weeks, including 20% CHO, 60% fat, and 20% protein	Exercise occurring after high-fat diet (treatment)	Passive avoidance task	Yes	Yes	Improved brain insulin signaling (PI3K/AKT/GSK-3β), reduced tau hyperphosphorylation.
Shi et al. (2018) [40]	Male C57BL/6 mice and SIRT3 mice (2-months old)	Exercise started at week 6 and continued for the remaining 6 weeks. Engaged in aerobic intermittent training, 30 min/day, 5 days/week. Intermittent exercise involved 4-min bursts at 80–85% of VO2max, with 2 min active recovery periods.	High-fat diet of 45% kcal fat, 20% kcal protein, and 35% kcal CHO for 12 weeks	Concurrent	Morris water maze	Yes	Yes	SIRT3 upregulation and improvement in antioxidative activity
Han et al. (2019) [41]	Six-week-old C57BL/6 mice	23 weeks of treadmill running, 30 min/day, 5 days/week, at 8 m/min.	23 weeks of high-fat diet ad libitum, involving 60% fat	Concurrent	Morris water maze	Yes	Yes	Reduced number of apoptotic cells and increased BDNF.
Mehta et al. (2019) [42]	Sprague–Dawley male rats	Running wheel access for 6 weeks, 25–30 min/day, 5 days/week.	15 days of high-fat diet (310 gm/kg Lard)	Exercise occurring after high-fat diet (treatment)	Passive avoidance and elevated plus maze	Yes	Yes	Reduction in neuroinflammatory markers (e.g., IL-1β, TNF-α).

## References

[1] DeSalvo K.B., Olson R., Casavale K.O. (2016). Dietary Guidelines for Americans. JAMA.

[2] Webster C.C., Swart J., Noakes T.D., Smith J.A. (2018). A Carbohydrate Ingestion Intervention in an Elite Athlete Who Follows a Low-Carbohydrate High-Fat Diet. Int. J. Sports Physiol. Perform..

[3] Noakes T.D., Windt J. (2017). Evidence that supports the prescription of low-carbohydrate high-fat diets: A narrative review. Br. J. Sports Med..

[4] Molteni R., Wu A., Vaynman S., Ying Z., Barnard R., Gómez-Pinilla F. (2004). Exercise reverses the harmful effects of consumption of a high-fat diet on synaptic and behavioral plasticity associated to the action of brain-derived neurotrophic factor. Neuroscience.

[5] Maesako M., Uemura K., Kubota M., Kuzuya A., Sasaki K., Asada M., Watanabe K., Hayashida N., Ihara M., Ito H. (2012). Environmental enrichment ameliorated high-fat diet-induced Aβ deposition and memory deficit in APP transgenic mice. Neurobiol. Aging.

[6] Tulving E. (1983). Elements of Episodic Memory.

[7] Mishkin M., Suzuki W.A., Gadian D.G., Vargha-Khadem F., Price T. (1997). Hierarchical organization of cognitive memory. Philos. Trans. R. Soc. B Boil. Sci..

[8] Poo M.-M., Pignatelli M., Ryan T.J., Tonegawa S., Bonhoeffer T., Martin K.C., Rudenko A., Tsai L.-H., Tsien R.W., Fishell G. (2016). What is memory? The present state of the engram. BMC Boil..

[9] Bliss T.V.P., Lømo T. (1973). Long-lasting potentiation of synaptic transmission in the dentate area of the anaesthetized rabbit following stimulation of the perforant path. J. Physiol..

[10] Bol’shakov V. (2001). Mechanisms of long-term synaptic depression in the hippocampus. Rossiiskii Fiziologicheskii Zhurnal Imeni I.M. Sechenova.

[11] Doyère V., Debiec J., Monfils M.-H., E Schafe G., E LeDoux J. (2007). Synapse-specific reconsolidation of distinct fear memories in the lateral amygdala. Nat. Neurosci..

[12] Whitlock J.R., Heynen A.J., Shuler M.G., Bear M.F. (2006). Learning induces long-term potentiation in the hippocampus. Science.

[13] Gruart A., Muñoz M.D., Delgado-García J.M. (2006). Involvement of the CA3-CA1 Synapse in the Acquisition of Associative Learning in Behaving Mice. J. Neurosci..

[14] Hao S., Dey A., Yu X., Stranahan A.M. (2016). Dietary obesity reversibly induces synaptic stripping by microglia and impairs hippocampal plasticity. Brain Behav. Immun..

[15] Valladolid-Acebes I., Merino B., Principato A., Fole A., Barbas C., Lorenzo M.P., Del Olmo N., Ruiz-Gayo M., Cano V., Garcia A. (2012). High-fat diets induce changes in hippocampal glutamate metabolism and neurotransmission. Am. J. Physiol. Metab..

[16] Karimi S.A., Salehi I., Komaki A., Sarihi A., Zarei M., Shahidi S. (2013). Effect of high-fat diet and antioxidants on hippocampal long-term potentiation in rats: An in vivo study. Brain Res..

[17] Wang Z., Fan J., Wang J., Li Y., Xiao L., Duan D., Wang Q. (2016). Protective effect of lycopene on high-fat diet-induced cognitive impairment in rats. Neurosci. Lett..

[18] Cano V., Valladolid-Acebes I., Hernández-Nuño F., Merino B., Del Olmo N., Chowen J.A., Ruiz-Gayo M. (2014). Morphological changes in glial fibrillary acidic protein immunopositive astrocytes in the hippocampus of dietary-induced obese mice. NeuroReport.

[19] Page K.C., Jones E.K., Anday E.K. (2014). Maternal and postweaning high-fat diets disturb hippocampal gene expression, learning, and memory function. Am. J. Physiol. Integr. Comp. Physiol..

[20] Molteni R., Barnard R., Ying Z., Roberts C., Gómez-Pinilla F. (2002). A high-fat, refined sugar diet reduces hippocampal brain-derived neurotrophic factor, neuronal plasticity, and learning. Neuroscience.

[21] Tozuka Y., Kumon M., Wada E., Onodera M., Mochizuki H., Wada K. (2010). Maternal obesity impairs hippocampal BDNF production and spatial learning performance in young mouse offspring. Neurochem. Int..

[22] Cassilhas R., Lee K., Fernandes J., Oliveira M., Tufik S., Meeusen R., De Mello M. (2012). Spatial memory is improved by aerobic and resistance exercise through divergent molecular mechanisms. Neuroscience.

[23] Van Praag H., Christie B.R., Sejnowski T.J., Gage F.H. (1999). Running enhances neurogenesis, learning, and long-term potentiation in mice. Proc. Natl. Acad. Sci. USA.

[24] Huang Y.-Q., Wu C., He X.-F., Wu D., He X., Liang F.-Y., Dai G.-Y., Pei Z., Xu G.-Q., Lan Y. (2018). Effects of Voluntary Wheel-Running Types on Hippocampal Neurogenesis and Spatial Cognition in Middle-Aged Mice. Front. Cell. Neurosci..

[25] Molteni R., Ying Z., Gomez-Pinilla F., Gómez-Pinilla F. (2002). Differential effects of acute and chronic exercise on plasticity-related genes in the rat hippocampus revealed by microarray. Eur. J. Neurosci..

[26] Dietrich M.O., Mantese C.E., Porciúncula L.O., Ghisleni G., Vinade L., Souza D.O., Portela L.V. (2005). Exercise affects glutamate receptors in postsynaptic densities from cortical mice brain. Brain Res..

[27] Bramer W.M., Rethlefsen M.L., Kleijnen J., Franco O.H. (2017). Optimal database combinations for literature searches in systematic reviews: A prospective exploratory study. Syst. Rev..

[28] Klein C., Jonas W., Iggena D., Empl L., Rivalan M., Wiedmer P., Spranger J., Hellweg R., Winter Y., Steiner B. (2016). Exercise prevents high-fat diet-induced impairment of flexible memory expression in the water maze and modulates adult hippocampal neurogenesis in mice. Neurobiol. Learn. Mem..

[29] Maesako M., Uemura K., Iwata A., Kubota M., Watanabe K., Uemura M., Noda Y., Asada-Utsugi M., Kihara T., Takahashi R. (2013). Continuation of Exercise Is Necessary to Inhibit High Fat Diet-Induced β-Amyloid Deposition and Memory Deficit in Amyloid Precursor Protein Transgenic Mice. PLoS ONE.

[30] Maesako M., Uemura K., Kubota M., Kuzuya A., Sasaki K., Hayashida N., Asada-Utsugi M., Watanabe K., Uemura M., Kihara T. (2012). Exercise Is More Effective than Diet Control in Preventing High Fat Diet-induced β-Amyloid Deposition and Memory Deficit in Amyloid Precursor Protein Transgenic Mice. J. Boil. Chem..

[31] Woo J., Shin K.O., Park S.Y., Jang K.S., Kang S. (2013). Effects of exercise and diet change on cognition function and synaptic plasticity in high fat diet induced obese rats. Lipids Heal. Dis..

[32] Noble E.E., Mavanji V., Little M.R., Billington C.J., Kotz C.M., Wang C. (2014). Exercise reduces diet-induced cognitive decline and increases hippocampal brain-derived neurotrophic factor in CA3 neurons. Neurobiol. Learn. Mem..

[33] Cheng J., Chen L., Han S., Qin L., Chen N., Wan Z. (2016). Treadmill Running and Rutin Reverse High Fat Diet Induced Cognitive Impairment in Diet Induced Obese Mice. J. Nutr. Health Aging.

[34] Kang E., Koo J., Jang Y., Yang C., Lee Y., Cosio-Lima L.M., Cho J. (2016). Neuroprotective Effects of Endurance Exercise against High Fat Diet-Induced Hippocampal Neuroinflammation. J. Neuroendocr..

[35] Kim T.-W., Choi H.-H., Chung Y.-R. (2016). Treadmill exercise alleviates impairment of cognitive function by enhancing hippocampal neuroplasticity in the high-fat diet-induced obese mice. J. Exerc. Rehabil..

[36] Park H.S., Cho H.S., Kim T.W. (2018). Physical exercise promotes memory capability by enhancing hippocampal mitochondrial functions and inhibiting apoptosis in obesity-induced insulin resistance by high fat diet. Metab. Brain Dis..

[37] Cheng M., Cong J., Wu Y., Xie J., Wang S., Zhao Y., Zang X. (2018). Chronic Swimming Exercise Ameliorates Low-Soybean-Oil Diet-Induced Spatial Memory Impairment by Enhancing BDNF-Mediated Synaptic Potentiation in Developing Spontaneously Hypertensive Rats. Neurochem. Res..

[38] Jeong J.-H., Koo J.-H., Cho J.-Y., Kang E.-B. (2018). Neuroprotective effect of treadmill exercise against blunted brain insulin signaling, NADPH oxidase, and Tau hyperphosphorylation in rats fed a high-fat diet. Brain Res. Bull..

[39] Jeong J.-H., Kang E.-B. (2018). Effects of treadmill exercise on PI3K/AKT/GSK-3β pathway and tau protein in high-fat diet-fed rats. J. Exerc. Nutr. Biochem..

[40] Shi Z., Li C., Yin Y., Yang Z., Xue H., Mu N., Wang Y., Liu M., Ma H. (2018). Aerobic Interval Training Regulated SIRT3 Attenuates High-Fat-Diet-Associated Cognitive Dysfunction. BioMed Res. Int..

[41] Han T.-K., Leem Y.-H., Kim H.-S. (2019). Treadmill exercise restores high fat diet-induced disturbance of hippocampal neurogenesis through β2-adrenergic receptor-dependent induction of thioredoxin-1 and brain-derived neurotrophic factor. Brain Res..

[42] Mehta B.K., Singh K.K., Banerjee S. (2019). Effect of exercise on type 2 diabetes-associated cognitive impairment in rats. Int. J. Neurosci..

[43] Blitzer R.D. (2005). Teaching resources. Long-term potentiation: Mechanisms of induction and maintenance. Sci. STKE.

[44] Sweatt J.D. (1999). Toward a Molecular Explanation for Long-Term Potentiation. Learn. Mem..

[45] Buonarati O.R., Hammes E.A., Watson J.F., Greger I.H., Hell J.W. (2019). Mechanisms of postsynaptic localization of AMPA-type glutamate receptors and their regulation during long-term potentiation. Sci. Signal..

[46] Sacktor T.C., Fenton A.A. (2018). What does LTP tell us about the roles of CaMKII and PKMzeta in memory?. Mol. Brain.

[47] Baltaci S.B., Mogulkoc R., Baltacim A.K. (2019). Molecular Mechanisms of Early and Late LTP. Neurochem. Res..

[48] Park M. (2018). AMPA Receptor Trafficking for Postsynaptic Potentiation. Front. Cell. Neurosci..

[49] Mizuno M., Yamada K., He J., Nakajima A., Nabeshima T. (2003). Involvement of BDNF Receptor TrkB in Spatial Memory Formation. Learn. Mem..

[50] Mizuno M., Yamada K., Olariu A., Nawa H., Nabeshima T. (2000). Involvement of Brain-Derived Neurotrophic Factor in Spatial Memory Formation and Maintenance in a Radial Arm Maze Test in Rats. J. Neurosci..

[51] Sleiman S.F., Henry J., Al-Haddad R., El Hayek L., Haidar E.A., Stringer T., Ulja D., Karuppagounder S.S., Holson E.B., Ratan R.R. (2016). Exercise promotes the expression of brain derived neurotrophic factor (BDNF) through the action of the ketone body β-hydroxybutyrate. eLife.

[52] Qiao S., Peng R., Yan H., Gao Y., Wang C., Wang S., Zou Y., Xu X., Zhao L., Dong J. (2014). Reduction of Phosphorylated Synapsin I (Ser-553) Leads to Spatial Memory Impairment by Attenuating GABA Release after Microwave Exposure in Wistar Rats. PLoS ONE.

[53] John J.P.P., Sunyer B., Höger H., Pollak A., Lubec G. (2009). Hippocampal synapsin isoform levels are linked to spatial memory enhancement by SGS742. Hippocampus.

[54] Vaynman S., Ying Z., Gómez-Pinilla F., Gómez-Pinilla F. (2004). Exercise induces BDNF and synapsin I to specific hippocampal subfields. J. Neurosci. Res..

[55] Jovanovic J.N., Czernik A.J., Fienberg A.A., Greengard P., Sihra T.S. (2000). Synapsins as mediators of BDNF-enhanced neurotransmitter release. Nat. Neurosci..

[56] Rossi C., Angelucci A., Costantin L., Braschi C., Mazzantini M., Babbini F., Fabbri M.E., Tessarollo L., Maffei L., Berardi N. (2006). Brain-derived neurotrophic factor (BDNF) is required for the enhancement of hippocampal neurogenesis following environmental enrichment. Eur. J. Neurosci..

[57] Dupret D., Revest J.-M., Koehl M., Ichas F., De Giorgi F., Costet P., Abrous D.N., Piazza P.V. (2008). Spatial Relational Memory Requires Hippocampal Adult Neurogenesis. PLoS ONE.

[58] França T.F.A., Bitencourt A.M., Maximilla N.R., Barros D.M., Monserrat J.M. (2017). Hippocampal neurogenesis and pattern separation: A meta-analysis of behavioral data. Hippocampus.

[59] Lindqvist A., Mohapel P., Bouter B., Frielingsdorf H., Pizzo D., Brundin P., Erlanson-Albertsson C., Erlanson-Albertsson C., Erlanson-Albertsson C. (2006). High-fat diet impairs hippocampal neurogenesis in male rats. Eur. J. Neurol..

[60] Lee J., Duan W., Mattson M.P. (2002). Evidence that brain-derived neurotrophic factor is required for basal neurogenesis and mediates, in part, the enhancement of neurogenesis by dietary restriction in the hippocampus of adult mice. J. Neurochem..

